# Efficacy of Virtual Preparation Simulators Compared to Traditional Preparations on Phantom Heads

**DOI:** 10.3390/dj12080259

**Published:** 2024-08-14

**Authors:** Lea Stoilov, Fabian Stephan, Helmut Stark, Norbert Enkling, Dominik Kraus, Milan Stoilov

**Affiliations:** 1 Department of Prosthodontics, Preclinical Education and Dental Materials Science, University of Bonn, 53111 Bonn, Germany; 2 Department of Reconstructive Dentistry and Gerodontology, School of Dental Medicine, University of Bern, 3012 Bern, Switzerland

**Keywords:** dental education, digital dentistry, SIMtoCARE, virtual simulation, HVRS

## Abstract

Background: Virtual simulators are increasingly being introduced in dental education. This study investigates whether virtual simulators offer comparable or superior educational efficacy when compared to traditional phantom simulators. Materials and Methods: Participants were randomly allocated into groups: Virtual Preparation (SIM; n = 30) and Traditional Preparation (FRA; n = 30). Students were tasked with preparing tooth 36 for a full-cast crown during free practice for four days. Faculty staff provided feedback to both groups. Examinations were administered and graded by three examiners (preclinical and clinical consultants and a dental surgery consultant). Additionally, a survey was conducted to assess each training concept. Results: The FRA group achieved significantly better grades in the preparation exam evaluations by all three examiners, compared to the SIM group. Interrater reliability showed only moderate agreement, with the clinical examiner giving better grades than the other two. The questionnaire results indicate that while participants managed with the virtual system, they preferred the analog system for exams and patient preparation. Conclusion: Virtual simulators do not seem to be as good when it comes to practicing for a preparation exam or clinical preparation, especially for unexperienced students. However, they still appear to be useful as an additional tool for introducing students to the topic of preparation.

## 1. Introduction

The progress of digital dentistry is increasingly making its way into dental education and training, where digital workflows are replacing analog methods. A classic example of this is the replacement of conventional impressions and lost-wax casting in practical prosthodontic courses with intraoral scans and chairside milling of restorations. This shift allows students to familiarize themselves with modern technologies, while ensuring a standardized quality of work by minimizing variations due to deficient material processing. The use of chairside systems, such as the CEREC^®^ system (Dentsply Sirona^©^, Bensheim, Germany), marked the beginning of the digitization of dental education. In the preclinical environment, it is employed as a self-assessment tool (PrepCheck^®^, Dentsply Sirona^©^, Bensheim, Germany) and provides feedback on students’ preparations. However, conventional phantom heads with attachable and detachable models and interchangeable plastic teeth are still required. Thus, this concept occupies a transitional position between the traditional training approach and an innovative, purely digital approach.

Based on experience, dental preparation for crown placement poses a challenging learning task for students in the foundational years of dental education. The learning process is time-consuming, and the initial results can be frustrating for both students and instructors. To address this issue, digital evaluation systems, such as PrepCheck^®^ (Dentsply Sirona^©^, Bensheim, Germany), Compare (Planmeca^©^ Oy, Helsinki, Finland), or Dental Teacher™ (KaVo^©^ Dental, Biberach, Germany), have been implemented. These systems are seen as software extensions within the corresponding scanning system, assisting students in learning preparation techniques and, additionally, practicing digital impressions. In the context of Computer-Assisted Learning (CAL), these systems can highlight initial rough errors without contact with the faculty staff [1,2]. Students can learn autonomously or through self-directed learning, engaging with the faculty staff at a higher skill level in further inquiries. Park et al. [3] described them as “useful” for bridging the gap between learning styles and supporting students’ self-assessment. Furthermore, students appreciate immediate, objective, and, particularly, visual feedback on their deficiencies [4,5]. Rosenberg et al. [6] found that computer-assisted training is even superior, or at least equivalent, to traditional methods. In a previous study by the authors, it was also demonstrated that these systems appear to be equivalent, with the limitation that they cannot provide feedback on how to approach tooth preparation, problem-solving, and ergonomics [7].

Although traditional phantom heads still represent the gold standard in training and have been supplemented by modern self-evaluation systems, they cannot precisely simulate the patient cases encountered in clinical practice. In addition, anatomical structures such as a moving tongue can only be inadequately mimicked. Furthermore, dental education standards expect the teaching of increasingly complex procedures or competencies that can no longer be adequately taught using phantom heads, and might require the use of modern digital systems [8].

Virtual systems (Haptic Virtual Reality Simulation, HVRS), such as the “Dente” (SIMtoCARE, Vreeland, The Netherlands), can now virtually simulate most dental treatments. This includes practicing simple measures as well as operative or prosthetic treatment steps. Additionally, implant placement or even local anesthesia can now be practiced. Furthermore, patient-specific cases can be incorporated after digitalization and practiced. This is particularly useful for simulating treatment procedures in advance, before they are performed on real patients. Undergraduate students initially learn operative exercises, such as preparing cavities and, especially, teeth for receiving full crowns. Students could gain their first experience in handling the handpiece and dental mirror. All of this occurs in a more playful environment, one similar to a video game. They are gradually introduced to dental practice through the virtual systems, starting with simple, less complex exercises for hand–eye coordination. This approach could offer potential advantages for beginners, as initial attempts to adequately prepare a tooth are often challenging for both students and supervising faculty. The load on the faculty staff could be reduced at this level, as students autonomously perform appropriate exercises and receive virtual feedback. Some studies support virtual simulators as supplements in the preclinical environment, as students seem to perform well with a virtual simulator. However, these systems cannot yet replace phantom heads [9,10,11].

In universities and dental education, traditional phantom heads with plastic models and teeth continue to be regarded as the gold standard. They are used both in practical courses and in corresponding exams or state examinations. However, practice time is often very limited and restricted to class times, and support is provided by the faculty staff. Students often criticize this situation and demand the opportunity for free practice time [3]. Both points require additional staffing, which in turn increases costs. The possibility of practicing autonomously in a dental simulation laboratory that is accessible around the clock could provide a solution. Students would log in with a personalized identifier, allowing tracking of which student worked at what time and for how long. HVRS systems could be used for this purpose and enable free practice. The aspect of sustainability should also be considered. HVRS systems generate less plastic waste, require no compressed air and no suction, and do not waste water that subsequently needs to be treated.

This study aims to examine whether HVRS systems can be considered to be equivalent to phantom heads. Specifically, we investigated their effectiveness in teaching crown preparation and preparing students in an auto-didactic environment (free practice time) for a crown preparation exam.

## 2. Materials and Methods

The present investigation was conducted at the Department of Prosthetic Dentistry, Propaedeutics and Materials Science, Center for Dental, Oral, and Maxillofacial Diseases, at the University Hospital Bonn. A total of 60 dental students (50 female and 10 male) participated in this voluntary study.

Participants were contacted and recruited using printed flyers and social media. Informed consent was obtained from all students prior to the initiation of the study. Ethical approval was obtained from the ethics committee of the medical faculty of the University of Bonn (number 159/20).

Inclusion criteria were defined as follows:

- Students studying according to the new licensing regulations for dentists in Germany;
- Students in the 1st to 4th semester (equal skill level regarding tooth preparation);
- Students without preparation experience;
- Students who had not participated in the fixed prosthodontics preclinical course.

Upon registration, participants were randomly assigned a number from “1” to “60” to ensure anonymous group assignment and evaluation. All participants first received an introductory lecture to cover theoretical basics in preparation shapes and requirements, materials science, ergonomics, and the operation of respective analog and digital simulators. The study’s objective, detailed procedures, and tasks were explained, and any remaining questions were clarified to ensure all participants had the same level of understanding.

After the introductory lecture, a preliminary exercise was conducted to ensure an even distribution of initially skilled and less skilled participants within each group. All participants had to prepare tooth 36, without neighboring teeth, on the ANKA-4V dental model (Frasaco^©^, Tettnang, Germany), for a full-cast crown within two hours. Each student had only one attempt; no repetition of the exercise was allowed if a tooth was improperly prepared. Each participant was provided with a phantom workstation with accessories, a high-speed handpiece (Expertmatic Lux E25L, KaVo Dental^©^, Biberach, Germany), and the practice tooth. The ANKA-4V dental models, the phantom mask (Frasaco^©^, Tettnang, Germany), basic dental instruments (mirror, probe, and tweezers), and the requested preparation burs were part of the course materials from a previous course and were brought by the participants. They were given guidelines for ideal preparation and a model of the ideal preparation. The preparation margin was to be 0.5 mm supragingival with a preparation angle of 4–6° or a convergence angle of 8–12°, respectively. The circular substance removal was defined as 0.5 mm and the occlusal as 1 mm, with an aesthetic bevel and functional bevel of 45°. These specifications also applied to the subsequent free practice time and the preparation exam at the end of the study.

The prepared teeth were archived in a blinded manner in a box marked with the corresponding student number, allowing for an anonymous evaluation of the tooth preparations by a preclinical senior physician from the Department of Prosthetic Dentistry, Propaedeutics and Materials Science at the Center for Dental, Oral, and Maxillofacial Diseases, University of Bonn. The examiner could choose from the grades “good” = “+”, “satisfactory” = “0”, and “poor” = “−”. This allowed the assessment of the students’ ability to execute a novel and complex practical task. Based on this, participants were evenly divided into the Frasaco group (FRA) (n = 30) and the SIMtoCare group (SIM) (n = 30).

**Figure 1:**
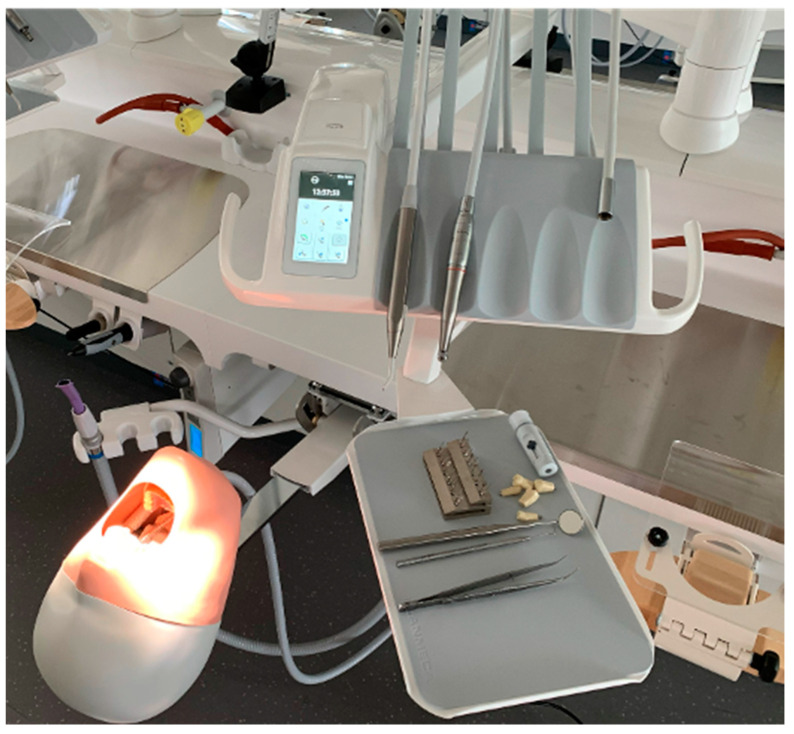
Phantom simulation unit by Planmeca^©^ (Helsinki, Finland) with mounted phantom head and model (Frasaco^©^, Tettnang, Germany).

**Figure 2:**
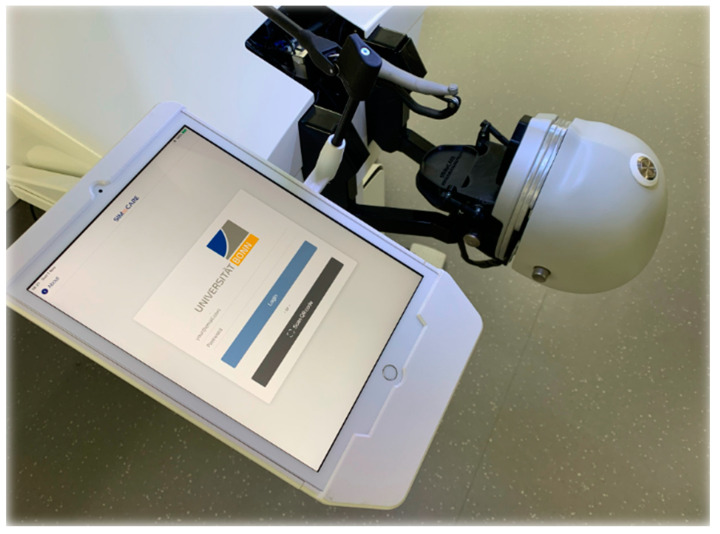
“Dente” simulator by SIMtoCARE (Vreeland, The Netherlands) with tablet, phantom head, and handpiece.

After group assignment, students were given the opportunity to improve their practical skills through free practice and enhance their preparation of tooth 36 for a full-cast crown. The FRA group worked with the ANKA-4V dental models (Frasaco^©^, Tettnang, Germany) and an analog simulation workstation (Figure 1), as described before. The SIM group performed the same training on the virtual preparation simulator “Dente” (SIMtoCARE, Vreeland, The Netherlands) (Figure 2). Each group had a total of 32 practice hours over four working days (8 h per day). On the fifth day, the preparation exam was conducted.

During free practice, students worked under realistic conditions with neighboring teeth. To ensure equal opportunities, the FRA group had unlimited access to consumables such as practice teeth, neighboring teeth, and gloves, allowing for an unlimited number of repetitions. Throughout the practice period, all participants could seek constructive feedback and suggestions for improvement from the preclinical course staff as often as needed. Additionally, participants could always view a plastic model of the master preparation of tooth 36 to spatially visualize the improvement suggestions.

### 2.1. Use of the “Dente” Simulator

At the start of the free practice period, all participants in the SIM group were first required to log into the “SIMtoCARE” (version number: 3779) learning software, using the provided tablet, with their assigned username and password. They then needed to select the “Prosthodontics” module and the “04-Crown” case. A dedicated course was created in the SIMtoCARE software specifically for this study. Each participant was assigned to this course, which contained only the “04-Crown” exercise, ensuring that only this exercise could be selected and trained. The recommended instruments (handpiece, mirror, and bur) were enabled, and all alternative instruments were hidden to avoid distractions or deviations.

Upon successful selection, the dental model and the instruments to be used were displayed in their original sizes on the three-dimensional screen. The user could interact with the virtual dental model and perform the necessary preparation on tooth 36, including tasks involving neighboring teeth and the opposing jaw, using the virtual representations of the instruments in real time. A physical handpiece, a handle on the device to move the dental mirror, and a physical foot pedal were available. Numerous supporting functions were also available, including height adjustment of the device, customizable magnification (1.0–4.5×) of the dental model, and the options to show/hide the opposing jaw, adjust the light angle, and display the percentage of enamel and dentin removed during preparation. Finally, the user could upload the result to the teaching staff by swiping right on the tablet and pressing the “Submit” button.

### 2.2. Preparation Exam and Evaluation

On the last day of the practice blocks for each group, a final preparation exam was conducted for all students, regardless of their practice group. All students had to prepare tooth 36 for a full-cast crown, using neighboring teeth, on the Frasaco model ANKA-4V (Frasaco^©^, Tettnang, Germany) within two hours under exam conditions. After the time expired, teeth 35, 36, and 37 were submitted, marked, and stored in the corresponding numbered box to ensure an anonymous, blinded evaluation by the teaching staff.

The prepared teeth were evaluated by three senior physicians from the Center for Dental, Oral, and Maxillofacial Diseases at the University of Bonn, each with at least 15 years of experience in student teaching. The evaluators included two senior physicians from the Department of Prosthetic Dentistry, Propaedeutics and Materials Science, one teaching in the preclinical and the other in the clinical study section, who regularly assess student preparations in their respective sections. The third examiner was a senior physician in oral surgery, a field in which student preparations are not assessed in dental education, providing a basis for an objective evaluation by reducing potential subjective influences such as familiarity effects.

The evaluators independently rated the preparations, conducting the assessments at different times and locations. They were provided only with the preparation results from the final exam and the opposing jaw model, along with a uniform evaluation form with predefined criteria validated in a previous study [7]. The grading criteria included possibility of crown retention, substance removal, preparation angle, bevel, surface roughness, potential undercuts, preparation margin, and lack of damage to neighboring teeth (Table 1). The evaluators could grade “Good” = “+”, “Average” = “0”, and “Poor” = “−”. A sum score was calculated for the final grade, with “+” equaling 5 points, “0” equaling 2.5 points, and “−” equaling −1 point, corresponding to a grading scale from 1 = “very good” to 5 = “deficient” (German grading scale) (35 to ≤7) (Table 1). For the criterion “No damage to neighboring teeth”, only “+” or “−” were available, with the examiner required to make a clear decision. Even minor damage to neighboring teeth was considered an iatrogenic injury and was marked negatively. The most important evaluation criterion was whether a crown could be retained on the tooth, with “Yes” or “No” as the options. If this criterion was rated negatively, 21 points were automatically deducted from the total score, making “4” (sufficient) the highest achievable grade.

### 2.3. Assessment of the Systems

After the performance assessment, all participants were asked to complete an evaluation form corresponding to their group (FRA or SIM) (Table 2). The evaluation aimed to determine how intuitive or complicated the operation of each method was and whether the students felt well-prepared for the final preparation exam through the respective training methods. Additionally, students were asked if they would be interested in using the respective training system in the future and to grade the system. The students could choose from grades “1” to “5” for statements A to F (Likert scale), with “1” = “strongly agree,” “2” = “agree,” “3” = “neither agree nor disagree,” “4” = “disagree,” and “5” = “strongly disagree.” For statement G, the answers “1” to “5” were assigned weights corresponding to the school grading system: “1” = “very good,” “2” = “good,” “3” = “satisfactory,” “4” = “sufficient,” and “5” = “poor.” Additionally, participants could write down suggestions for potential improvements in a free-text box.

### 2.4. Statistical Analysis

To calculate the statistically necessary number of participants for the present investigation, the power analysis previously described in Stoilov et al. [7] was used. The present authors assumed a type I error probability of 0.05, a standard deviation of 2.3 grades, and a minimally detectable effect size of 1.5 grades (dRMSSE = 0.25). With a power of 80%, a sample size of 35 per group was calculated. However, limited access to students led to a maximum sample size of 30 participants per group (60 participants total). Considering the maximum available sample size and maintaining the other parameters, a minimally detectable effect size of 1.5 grades (dRMSSE = 0.4) was calculated for this study (SPSS Statistics 27, IBM^®^, Armonk, NY, USA).

For statistical evaluation, the results from the aforementioned evaluation forms were first descriptively compiled using “Microsoft Excel” (version number: 16.78.3 (23102801)) (Microsoft^©^ Corporation, Redmond, WA, USA). Subsequently, statistical analysis and visualization of the results in box plot diagrams were conducted using the statistics software PRISM 10™ (GraphPad^©^ Software, Boston, MA, USA). The significance was tested using the Mann–Whitney U-test with an assumed significance level of α = 5%. The inter-rater reliability was determined using the Friedman test, Dunn’s multiple comparison test, and Fleiss’ Kappa.

## 3. Results

### 3.1. Group FRA versus Group SIM

**Figure 3:**
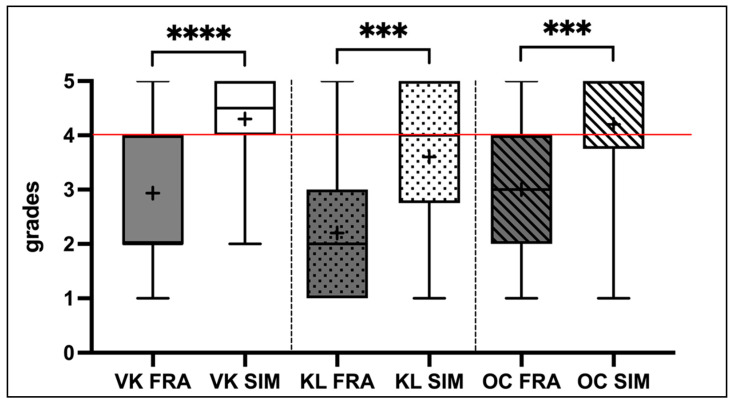
Box plot diagram of the grades from the preparation exam evaluated by the three examiners (VK = Preclinical Prosthetics, KL = Clinical Prosthetics, and OC = Oral Surgery) for participants in groups FRA and SIM. The “+” symbol indicates the arithmetic mean of the grades. The horizontal red line marks the passing threshold. Statistically significant differences between the respective groups are visually represented by asterisks “*”, with significance levels indicated as *p* < 0.001 (“***”), and *p* < 0.0001 (“****”).

All 60 participants completed the preliminary exercise, the free practice sessions, the final exam, and the evaluation form. No drop-outs were documented. Table 3 shows the scores given by the three evaluators and the score distribution within each group (mean, SD, and median). The preclinical evaluator (Prosthodontics, VK) graded the participants in the Frasaco group (FRA) with a median score of 2 (“good”; Mean: 2.93 ± 1.26), which was better than those in the SIMtoCARE group (SIM), who received a median score of 4.5 (“sufficient”; Mean: 4.30 ± 0.85). Similarly, the clinical evaluator (Prosthodontics, KL) gave comparable grades, with a median score of 2 (“good”; Mean: 2.20 ± 1.24) for the FRA group and 4 (“sufficient”; Mean: 3.60 ± 1.43) for the SIM group. The oral surgery evaluator (Surgery, OC) rated both groups one grade lower on average than the other two raters. He assigned a median score of 3 (“satisfactory”; Mean: 3.0 ± 1.23) for the FRA group and 5 (“poor”; Mean: 4.2 ± 1.06) for the SIM group. Statistical analysis assumed a normal distribution of the grades. The evaluation from each examiner showed significantly better scores in the Frasaco groups (VK: *p* < 0.0001; KL: *p* = 0.0003; OC: *p* = 0.0002) when applying the Mann–Whitney U test (Table 4 and Figure 3).

### 3.2. Interrater Reliability

**Figure 4:**
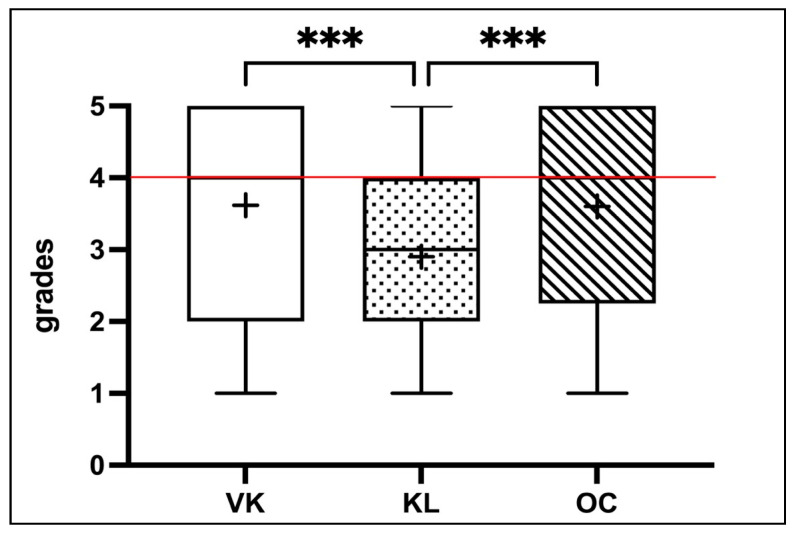
Box plot diagram illustrating the grade distributions for the preparation exam as evaluated by the three examiners. The “+” symbol denotes the arithmetic mean of the grades for each examiner. The horizontal red line represents the passing threshold. Statistically significant differences between groups are indicated by asterisks “*”. The number of asterisks corresponds to the *p*-value significance level: *p* < 0.001 (“***”).

Each examiner independently and blindly evaluated a total of 60 preparations (FRA group, n = 30; SIM group, n = 30). The preclinical evaluator (VK) gave a median score of 4 (“sufficient”; Mean: 3.62 ± 1.26), while the clinical evaluator (KL) gave a median score of 3 (“satisfactory”; Mean: 2.90 ± 1.50). The oral surgery evaluator assigned a median score of 4 (“sufficient”; Mean: 3.60 ± 1.26), which was equivalent to that of the VK evaluator (Table 5). For the given grade distribution, no normal distribution was assumed. Therefore, the Friedman test was used to check for differences in grading. With an assumed significance level of α = 5%, a significant difference (Friedman test statistic: 32.27; *p* < 0.0001) in grading among the three evaluators (VK mean rank = 2.25; KL mean rank = 1.54; OC mean rank = 2.21) was found (Figure 4). The calculated Fleiss’ Kappa, a measure of inter-rater agreement, showed a moderate agreement, with κ = 0.3 according to Altman (Table 6). The subsequent post-hoc analysis (Dunn’s Multiple Comparison Test) allowed targeted examination for statistically significant differences between the ratings of two evaluators. A significantly better rating by the clinical prosthodontics evaluator (KL) (mean rank = 1.54) compared to the preclinical prosthodontics evaluator (mean rank = 2.25) and the oral surgery evaluator (mean rank = 2.21) was identified (z = 3.88; *p* = 0.0003/z = −3.65; *p* = 0.0009). This represented a medium effect size, according to Cohen, with r = 0.35 and r = 0.33, respectively. However, there were no statistically significant differences between the grades given by the preclinical evaluator (VK) and the oral surgery evaluator (OC) (z = 0.23; *p* > 0.9999) (Table 7).

### 3.3. Assessment

**Figure 5:**
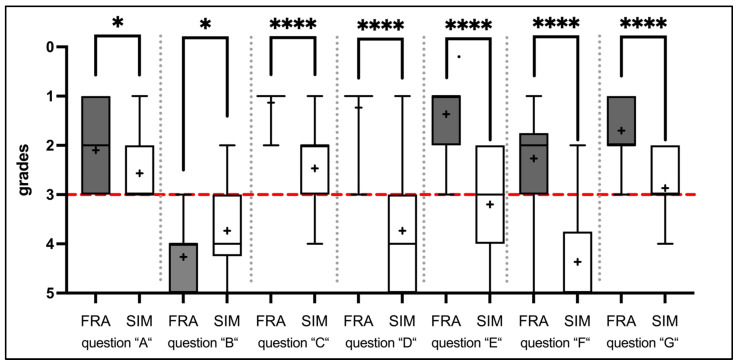
The horizontal red dashed line visualizes a “neutral” (grade 3) statement according to the Likert-type scale. The “+” symbol denotes the arithmetic mean of the grades for each examiner. Mean ± SEM were calculated and the Mann–Whitney U test (* = *p* < 0.05) was performed. “*” indicates significant statistical differences between the groups. The number of asterisks corresponds to the *p*-value significance level: *p* < 0.050 (“*”) and *p* < 0.0001 (“****”).

All study participants completed an evaluation form associated with their training system. The analysis of the questionnaires showed statistically significant differences for statements A-G. The corresponding evaluation results and score distribution, as well as the statistical analysis, can be found in Table 8 and Figure 5. No normal distribution was assumed for the given grade distributions, and the Mann–Whitney U test was used (α = 5%) to check for statistical significance. The answers to statement “H” focused particularly on technical improvements within the software and were, logically, asked exclusively to the SIM group participants. The responses are summarized in Table 9, with their frequency indicated.

Participants in the Frasaco group rated the operation of the analog simulation unit and the phantom head as more intuitive compared to the SIMtoCARE system (Statement “A”; “agree” versus “neutral”; *p* = 0.0209) (Table 8 and Figure 5). At the same time, both groups found the operation of their respective systems to be not complicated (Statement “B”; “neutral”; *p* = 0.0214). Overall, participants from the FRA group more strongly felt that their skills improved with their system than was the case in the SIM group (Statement “C”) (*p* < 0.0001), with the FRA group giving a median score of 1 (“fully agree”). The SIM group, however, gave a slightly lower score of “2” (“agree”). Interestingly, this contrasts somewhat with the ratings for statement “D”. Participants in the FRA group agreed that they wanted to continue practicing with the analog phantom head, while participants in the SIM group rejected further training with the virtual simulator (“fully agree” versus “disagree”; *p* < 0.0001). This opinion was reflected in the responses to statement “E”. Here, FRA group participants felt better prepared for the final preparation test by using the analog phantom head (“strongly agree”), compared to the SIM group participants who used the SIMtoCARE system (“neutral”) (*p* < 0.0001). Additionally, the confidence to prepare on a real patient was higher in the FRA group than in the SIM group (Statement “F”) (“agree” versus “strongly disagree”; *p* < 0.0001). Overall, both training systems were positively rated by the participants (Statement “G”). The analog phantom head received a median score of “2” (“good”), while the SIMtoCARE system received a median score of “3” (“satisfactory”; *p* < 0.0001).

## 4. Discussion

The education of dental students is a complex process that requires not only the acquisition of theoretical knowledge but also fine motor skills and a high level of hand–foot–eye coordination. Before invasive clinical procedures can be performed on patients, it is important to achieve sufficient competence through intensive use of preclinical practice, [12,13]. Traditionally, these skills are trained and learned using phantom heads and plastic models. Feedback is provided by the faculty, which is often described as a very subjective assessment of performance [14], leading to dissatisfaction among students [15]. To address this issue, digital evaluation systems (e.g., PrepCheck, Dental Teacher, and Romexis Compare) have been introduced in the context of digitalizing dental education. These systems overlay and compare students’ preparations with instructors’ master preparations, providing objective feedback [16]. It is even recommended to use these systems for calibrating instructors and as an adjunct in assessing preparation exams [7]. In a previous study by the authors, the effectiveness of these systems was investigated [7]. The results showed no significant difference in the performance of students who received faculty feedback versus feedback from the preparation assistants. However, students generally positively perceived the use of these digital systems. The primary function of these systems lies in generating free practice time, during which no feedback from a tutor or instructor is necessary, allowing students to learn and improve their skills autonomously [3]. This reduces the workload and creates more practice time in a phase where it is urgently needed [2].

As the next step in the digitalization of preclinical dental education, haptic virtual reality simulators (HVRS) are increasingly being adopted [17]. Advances in haptic technology have changed the perception of virtual reality (VR) and particularly of HVRS. The haptic experience in the virtual environment (VE) represents a bidirectional flow of information between the user and virtual reality, transmitting forces, vibrations, or movements to the user through a haptic interface and thus simulating tactile and kinesthetic sensations [18,19]. HVRS fundamentally change the interaction with virtual objects by providing a realistic sense of touch and feel [20], which is crucial for learning fine dental skills. The simulation experience is almost entirely transferred to the virtual world, eliminating the need for plastic teeth or real handpieces. Research in this field has intensified in recent years, demonstrating the supportive benefits of these systems in training for dental restorative procedures [21,22,23,24]. Consequently, more dental schools are implementing these HVR systems [25,26].

The present study aimed to investigate the effectiveness of virtual simulators (HVRS) for learning dental preparation (Prosthodontics) during the preclinical phase of dental education, in comparison to traditional learning using analog phantom heads. For this purpose, the preparation of tooth 36 for a full-cast crown was performed at an analog phantom workstation and on the Dente (SIMtoCARE) during a free practice period. A preparation exam was then conducted to determine the students’ progress.

The performance of students in the FRA group (analog simulation workstation) was significantly better than that of the SIM group (Dente, SIMtoCARE) (Figure 3). All three examiners rated the preparations of the FRA group two grades higher on average (VK: 2 vs. 4.5; KL: 2 vs. 4; OC: 3 vs. 5). Looking at the absolute grade distribution (Table 3), 50% and 53% of the participants in the SIM group received lower grades from examiners VK and OC, respectively, compared to the analog FRA group, and would have even failed the preparation exam. The rating from examiner KL was slightly better, but still showed a high failure rate of 37%. In contrast, the analog FRA group received significantly better ratings. The grade distribution reversed in this group, with the examiners awarding a grade of “2” to 50% (VK), 40% (KL), and 37% (OC), attesting to good performance. Ultimately, 33% even achieved a grade of “very good” (“1”) from examiner KL. Based on this data, the “Dente” (SIMtoCARE) alone does not seem suitable for preparing inexperienced students for a preparation exam on the phantom.

In a similar study, Arora et al. [27] also concluded that practice on an analog phantom head leads to better results than preparation exercises on HVRS. That study used the Virteasy Simulator (Virteasy Dental^©^, Changé, France) in order to determine its equivalence to the phantom head. However, the participants did not have to take a final exam on the phantom; instead, four preparations were performed and evaluated sequentially. Only inexperienced second-year students were included, who prepared either only on the Virteasy or only on the phantom head. Although the initial preparations showed a slight difference favoring conventional preparation, it was not statistically significant. Interestingly, this changed significantly for the third and fourth preparations. Participants in the virtual preparation group contrastingly scored approximately 50/100 points, while the analog group scored around 80/100 points. This is likely due to the last two preparations being performed on more complex premolars and molars. Although these results suggest that HVRS may result in poorer performance in crown preparation training, Arora et al. [27] confirm the higher effectiveness of virtual simulators. They base this conclusion on the improved scores observed over time with the use of the simulator. This is also confirmed in a systematic review by Moussa et al. [24], who report improvement in crown preparation over time after using HVRS with or without instructor feedback. It appears that HVRS is more effective for experienced users. This is further evidenced in the study by Wang et al. [28], which investigated the use of HVRS for metal–ceramic crown preparation. Their study used the Simodont dental trainer (MOOG^©^, Nieuw-Vennep, The Netherlands). Both novices and residents performed preparations, which were then evaluated. Additionally, the required time and errors in the preparations (i.e., occlusal reduction, undercuts, and damage to neighboring teeth) were recorded. The results showed that novices took significantly more time, received poorer evaluations, and made more errors. The detection of these differences demonstrates the validity of the system. Both groups found working with the simulator very positive and even preferred virtual training to preparing on plastic teeth. Murbay et al. [29] also examined the Simodont dental trainer (MOOG^©^, Nieuw-Vennep, The Netherlands) and showed that students who entered the study with the Simodont were able to perform far more satisfactory preparations (in multiple areas), compared to the group that did not use the simulator, despite having the same level of competence.

Philipp et al. [22] showed opposing results in their study, which had a similar design to ours and also used the Dente from SIMtoCARE (Vreeland, The Netherlands). They found no significant difference between the analog (control group) and virtual (test group) groups. However, the participants were tasked with performing an access cavity for a pulpotomy, which, although an invasive procedure, is performed on a much smaller area. Therefore, the complexity of the exercise is lower than the complete preparation of a molar with neighboring teeth. Furthermore, the participants had already gained preparation experience on the analog simulator, so unlike in the present study (completely inexperienced students), the handling of the phantom head for the final exam was familiar and simplified. The small number of participants (n = 14) also limits its statistical power. Despite the positive results, Philipp et al. [22] see HVRS more as a bridge between preclinical training on the phantom and real clinical patients. The authors of the present study share this view and currently see HVRS as a tool complementary to conventional training. Both approaches have advantages and disadvantages and can complement each other in their capabilities. The literature review by Imran et al. [30] and the systematic reviews by Moussa et al. [24] and Koolivand et al. [31] confirm the positive effect of HVRS and the good conveyance of psychomotor skills in managing clinical situations. Moussa et al. [24] emphasize the importance of force-feedback-based 3D simulators compared to 2D-based systems without force-feedback. They also recommend a combination of instructor feedback and device feedback. Imran et al. [30] highlight the reduced cost factor, as no technical requirements for the building (e.g., water supply, drainage, and suction) are necessary, and there is no need for regular maintenance or buying plastic teeth. However, it should be noted that HVRS represents sensitive, highly developed technology that can also malfunction. Corresponding repairs could be costly and result in the system being unavailable. These costs could be reduced in the future if HVRS finds wider application.

However, even in the future, the analog phantom head will remain the gold standard for preparation exams. This is because the simulation of hard tissues, the ergonomics of working with a simulated patient, the use of a real handpiece with variable speed and actual water cooling, and the operation of a real treatment unit cannot currently be adequately represented virtually. Additionally, if a shift to purely virtual training were to occur in the future, there would still need to be a transition from purely virtual treatment training to real patient care. The student must then be able to perform a sufficient preparation that does not endanger the patient, regardless of how well they performed previously in the virtual space or with virtual patients. For this reason, only a combination of analog and virtual simulation can currently be recommended, as the student must familiarize themselves with and adapt to a real treatment environment (the tangible analog phantom patient) at some point, at the latest, just before treating real patients. In this context, additional aspects become apparent that are missing in virtual simulation (Dente, SIMtoCARE). Excessive pressure, insufficient water cooling, and blunt or overly fine instruments (diamonds) ultimately lead to overheating of the prepared tooth and pose a risk to its vitality. An analog plastic tooth reacts with charring and the corresponding smell, providing the student with feedback on this fatal error.

The strength of virtual simulation, on the other hand, lies fundamentally in its ability to simulate a wide variety of dental procedures with a single system. Considering the standard portfolio of the Dente (SIMtoCARE), for example, it offers manual dexterity exercises, restorative procedures such as caries excavation, access cavity preparation, and pocket depth measurement. Additionally, prosthetic preparation exercises, implantations, and the administration of block anesthesia are available. Since new cases can theoretically be added as needed, the possibilities are endless. Particularly in the area of caries excavation, the practice cases can be designed as interactive exercises in which students receive direct feedback from the software as to whether all the caries has been removed or if residual caries remains. It even indicates how much healthy tooth substance was damaged during the treatment. Compared to the analog phantom, such a situation is generally difficult to simulate, and if attempted (e.g., using a model with extracted teeth), it requires supervision by a supervising dentist. This highlights the self-learning aspect of virtual simulation [32,33,34]. Jasinevicius et al. [35] demonstrated that students who completed their preclinical training with a virtual simulator required only 20% of the time that an instructor needed for personal instruction. It is worth mentioning that virtual systems can import real individual patient cases following an intraoral scan by, for example, importing the data in PLY format, and then voxelizing them. This allows for the repeated practice of this individual situation until a safe and successful approach for future treatment can be implemented. This capability presents an intriguing option, especially in clinical dental education.

The authors believe that a practice scenario needs to be created in the sense of a “guided preparation” that shows students a path to safely perform a preparation. This principle is applied in other fields, such as aviation, particularly with respect to landing. Pilots receive a predetermined “glideslope” through the Instrument Landing System (ILS), which ensures that the runway is safely reached and the landing is successful. They receive constant feedback through the “localizer” about height and distance to the runway and whether the aircraft is, for instance, too far to the right or left of the runway. Analogous to this principle, a tooth to be prepared could consist of multiple colored layers. When the student begins to prepare, they would first expose the tolerance range. Then, they would see the color of the ideal preparation and can orient themselves to it until they expose the color of the master preparation. If they grind too deeply, an alert color (e.g., red) might indicate excessive substance removal. This way, direct feedback is given during the preparation process, no human supervision is required, and the whole exercise has a playful aspect that may enhance the user’s ambition.

The evaluation of the feedback forms highlights the differences between the two systems under investigation. Participants’ subjective perceptions of their training methods were captured using seven statements to be rated (A to G). Additionally, suggestions for improvement could be provided in an optional free-text format (Statement H).

Initially, participants found working with the Dente (SIMtoCARE) found their system to be less intuitive, compared to those in the Frasaco group (Statement A). This is further supported by the results for statement B, “The operation of the Dente or phantom head was complicated,” which showed a significantly better rating from participants in the FRA group compared to those in the SIM group. Despite the difference in ratings, the grade distribution indicates that the perceived complexity of the Dente is not clear-cut. While the Dente might initially appear less intuitive and more complicated compared to the phantom head, participants did not perceive it as opaque or particularly complex.

Additionally, participants in the FRA group reported a greater improvement in their practical skills, compared to responses from those in the SIM group (Statement C). However, a closer look at the ratings reveals that despite these differences, there is an overall positive development (60% positive, 27% neutral, 13% negative) in the practical skills of the SIM group participants as perceived subjectively.

Nonetheless, 76% of the SIM group participants felt that even after adequate practice with the system, they were not capable of safely preparing a tooth on a patient (Statement F). In contrast, 70% of the FRA group participants felt confident they could successfully perform this task in the future. The starkly negative response to Statement D, “I would like to continue practicing with the system used here in the phantom course”, by the SIM group (56% rated it 4 or 5, “disagree/strongly disagree”) compared to the positive response by the FRA group (97% rated it 1 or 2, “strongly agree/agree”) underscores the differing perspectives and attitudes of the two groups regarding the skills acquired and to be acquired, as well as their confidence in using the system. This is further emphasized by the final rating of each system used (Statement G), in which the Dente was rated lower on average (score “3”) compared to the phantom head (score “2”), albeit not as decisively.

Zafar et al. [11] reached similar conclusions in their study. They investigated the subjective perceptions of students who performed pulpotomies and crown preparations using both an HVRS system (Simodont, Nissin Dental^©^, Kyoto, Japan) and a conventional dental model. In total, 56% of participants agreed that the HVRS simulator improved their understanding of pulpotomies and crown preparations. However, participants generally felt more comfortable using the phantom head with a dental model, compared to the HVRS simulator. This could be attributed to the participants’ prior familiarity with the analog system, leading to a bias against the HVRS.

The fundamentally poorer evaluation of the SIMtoCARE system by the students in this study can be attributed to various factors (Statement H). Primarily, participants felt that the system did not yet meet the gold standard of the phantom head, which remains the benchmark for examination situations. The virtual dental models could be rotated 360°, allowing users perspectives that did not correspond to the real situation. The lack of representation of the opposing jaw and the cheeks during preparation, as well as the ability to zoom in excessively, contributed to the perception that the range of motion of the handpiece and the visibility of details were less realistic.

Interestingly, Arora et al. [27] view these aspects as advantages of the virtual system, suggesting that they even contribute to improving students’ skills.

Additionally, some students found the digital lighting and shadow effects unrealistic, conditions which could distort a realistic working view. Moreover, participants noted several issues with the SIMtoCARE system’s application. Initially, the rotation speed of the digital handpiece was fixed at 60,000 RPM, which made finishing the virtual teeth impossible. A finished surface and precise preparation margin are crucial for a seamless transition from tooth to crown. Additionally, during system use, there were technical complications: either the screen froze, or there were deficits in the integrity (“holes”) of the virtual teeth. In both cases, a system restart was required without saving progress.

Consequently, users’ trust in the system was significantly undermined, as evidenced by the negative ratings for Statement D, “I would like to continue practicing with the system used here in the phantom course”; Statement E, “The practice system used prepared me well for the preparation examination”; and Statement F, “With the practice method, I see myself able to safely prepare a tooth on a patient after sufficient practice” by participants in the SIMtoCARE group.

Wang et al. [28] conducted a similar survey and found contrasting results. They surveyed both the novice students and dental residents who participated in their study. Both groups provided consistently positive ratings in favor of the HVRS. They found the system more user-friendly and efficient compared to conventional methods. Furthermore, they believed that using the HVRS would improve learning in the course and felt motivated to continue training with it rather than with plastic teeth. The study highlights that the so-called novices had already had preparation experience with the phantom and had completed the phantom course in restorative dentistry. Along with the dental residents, this study essentially surveyed a more experienced group. This aligns with the observation that HVRS appears to be more effective and better-accepted among experienced users.

Even among the inexperienced users in our study, the evaluations of both systems (Statement G) showed only minimal differences. Users rated the HVRS slightly worse (“satisfactory” versus “agree”), though it still received a “satisfactory” rating. It is expected that with longer-term use and increased experience, overall favorable perception will improve.

In conclusion, it is important to mention that the inter-rater reliability was investigated. Unlike in Stoilov et al. [7], an additional rater from the surgical field was included to achieve greater objectivity. However, the results indicate a significant difference in grading, with only moderate agreement (Fleiss’ Kappa; κ = 0.3). Two of the raters (VK and OC) were more similar in their evaluations, giving significantly lower grades compared to the rater KL. This was unexpected, as rater KL is responsible for assessing and grading preparations on actual patients. In contrast, the other raters are more accustomed to dealing with preclinical students and might be used to seeing lower-quality preparations. This situation further emphasizes the need for an objective evaluation system independent of human variability, one which cannot be affected by fluctuations in daily performance. In this context, the implementation and testing of evaluation software within the HVRS should be considered and investigated.

## 5. Conclusions

Considering the limitations of this study, it can be concluded that haptic feedback virtual simulators (HVRS) are not currently capable of training inexperienced students for a preparation exam, compared to the traditional phantom head. Training on the virtual simulator resulted in significantly lower scores on the preparation exam. Inexperienced students prefer working with the analog phantom head, even though they initially find the use of the HVRS manageable and subjectively perceive an improvement in their performance. From the authors’ perspective, a combination of both systems is necessary for future dental education to encompass both virtual and real-world training. For advanced students who wish to refine their skills in a more intensively simulated environment or engage in individual practice with patient cases, HVRS offers an efficient and cost-effective option.

## Figures and Tables

**Table 1:** Criteria assessed for evaluation of teeth prepared during the exam. Examiners had the option to rate with “+” (yes), “−” (no) or “0” (neutral). A total grade from 35–0 points, based on responses rated 1–5, could be awarded.

Assessed Criterion	Score	
Is a crown attachable?	Yes/No	
Substance removal?	Circular	Occlusal
	+/− or “0”	+/− or “0”
Damage to adjacent teeth?	+/−	
Finish line quality?	+/− or “0”	+/− or “0”
Preparation angle? (Undercuts? Conicity?)	+/− or “0”	+/− or “0”
Surface roughness?	+/− or “0”	+/− or “0”
Grade scale	Total grade	
35 to 0|1 to 5		

**Table 2:** Questions handed out to the participants at the end of the study. Participants had the opportunity to choose between “1” (strongly agree) and “5” (strongly disagree).

Statement		1	2	3	4	5
A	“Operating the Dente or Phantom simulator was simple”					
B	“Operating the Dente or the Phantom simulator was complex”					
C	“Practicing on the Dente or the Phantom simulator improved my skills”					
D	“In the phantom course, I would like to continue training with the system I used in this study”					
E	“The training system I used has prepared me well for the preparation exam”					
F	“With the training system and sufficient practice, I feel capable of preparing a tooth on a patient”					
G	“Please evaluate the training system you used”					
H	“What would you like to improve?”					

**Table 3:** Tabular representation of the absolute grade frequency, median, mean, and standard deviation (SD) of the performance evaluations by the three examiners (VK = Preclinical, KL = Clinical, and OC = Oral Surgery) for the respective participant groups FRA and SIM.

	Grade 1	Grade 2	Grade 3	Grade 4	Grade 5	Median	Mean ± SD
VK FRA	1	15	5	3	6	2	2.93 ± 1.26
VK SIM	0	1	4	10	15	4.5	4.30 ± 0.85
KL FRA	10	12	2	4	2	2	2.20 ± 1.24
KL SIM	4	3	5	7	11	4	3.60 ± 1.43
OC FRA	2	11	7	5	5	3	3 ± 1.23
OC SIM	1	1	5	7	16	5	4.20 ± 1.06

**Table 4:** A tabular representation of the rank statistics (mean rank and rank sum) for the grading of group FRA and group SIM by all three examiners (VK = Preclinical, KL = Clinical, and OC = Oral Surgery), including the calculated U-value and the associated *p*-value from the Mann–Whitney U-test to assess significant differences in participant grading. The effect size (r) according to Cohen is also indicated.

Rank Statistics				Mann–Whitney U-Test		
	n	Mean Rank (M_Rang_)	Rank Sum	U	Exact *p*-Value	r
VK FRA	30	21.75	652.5	187.5	<0.0001	0.52
VK SIM	30	39.25	1178			
KL FRA	30	22.7	681	216	0.0003	0.46
KL SIM	30	38.3	1149			
OC FRA	30	22.55	676.5	211.5	0.0002	0.47
OC SIM	30	38.45	1154			

**Table 5:** Presentation of the absolute grade distribution, median, mean, and standard deviation (SD) of the performance assessments by the three examiners for all participants.

	Grade 1	Grade 2	Grade 3	Grade 4	Grade 5	Median	Mean ± SD
Preclinic (VK)	1	16	9	13	21	4	3.62 ± 1.26
Clinic (KL)	14	15	7	11	13	3	2.90 ± 1.50
Oral surgery (OC)	3	12	12	12	21	4	3.60 ± 1.29

**Table 6:** Presentation of the rank statistics (mean rank and rank sum) of the grades assigned by the three examiners, along with the calculated test statistic corrected for ties and the associated *p*-value from the Friedman test to assess significant differences in the grading of participants across all groups. Fleiss’ Kappa (κ) is also included.

Rank Statistics				Friedman Test		Fleiss’ Kappa
	n	Mean Rank (M_Rang_)	Rank Sum	Friedman Test Statistic (Corrected)	Asymptotic *p*-Value	κ
Preclinic (VK)	60	2.25	135	32.27	0.0002	0.3
Clinic (KL)	60	1.54	92.5			
Oral surgery (OC)	60	2.21	132.5			

**Table 7:** Presentation of the test statistics and z-values calculated by Dunn’s Multiple Comparison Test to evaluate significant differences in grading between the three examiners. Additionally, uncorrected *p*-values and Bonferroni-corrected *p*-values, along with the effect size (r) according to Cohen for significant differences, are provided.

Dunn’s Multiple Comparison Test					
	Test Statistics	z-Value	Asymptotic *p*-Value (Not Corrected)	Asymptotic *p*-Value (Corrected)	r
VK vs. KL	0.71	3.88	0.0001	0.0003	0.35
VK vs. OC	0.04	0.23	0.8195	>0.9999	
KL vs. OC	−0.67	−3.65	0.0003	0.0009	0.33

**Table 8:** Tabular summary of the frequency distribution, median, mean, and standard deviation (SD) for each statement (A to G) on the evaluation form, reported by participants in both the FRA and SIM groups.

	Grade 1	Grade 2	Grade 3	Grade 4	Grade 5	Median	Mean ± SD
	“Strongly Agree”	“Agree”	“Neutral”	“Disagree”	“Strongly Disagree”		
Statement A: FRA	8	11	11	0	0	2	2.10 ± 0.80
Statement A: SIM	1	11	18	0	0	3	2.57 ± 0.57
Statement B: FRA	0	0	6	10	14	4	4.27 ± 0.78
Statement B: SIM	0	1	13	9	7	4	3.73 ± 0.87
Statement C: FRA	26	4	0	0	0	1	1.13 ± 0.35
Statement C: SIM	2	16	8	4	0	2	2.47 ± 0.82
Statement D: FRA	24	5	1	0	0	1	1.23 ± 0.50
Statement D: SIM	1	3	9	7	10	4	3.73 ± 1.14
Statement E: FRA	20	9	1	0	0	1	1.37 ± 0.56
Statement E: SIM	0	10	6	12	2	3	3.20 ± 1.00
Statement F: FRA	7	14	4	4	1	2	2.27 ± 1.08
Statement F: SIM	0	1	6	4	19	5	4.37 ± 0.93
Statement G: FRA	10	19	1	0	0	2	1.70 ± 0.54
Statement G: SIM	0	8	18	4	0	3	2.87 ± 0.63

**Table 9:** Tabular summary of criticisms and suggestions for improvement from the SIM group, including their absolute frequencies.

Free-Text Responses SIMtoCARE	Absolute Frequency
“The virtual model should not be able to rotate 360°”	10
“The speed must be adjustable”	7
“System crashes during use: frozen screen and “holes” in the teeth”	6
“The upper jaw and cheeks must also be displayed to simulate a more realistic situation”	4
“The light is unrealistic”	2
“The display resolution should be higher”	2
“Finishing the virtual teeth is not possible”	1
“You can zoom in too much”	1

## Data Availability

The original contributions presented in the study are included in the article.
